# Barriers and Motivators to Weight Loss in People With Obesity

**DOI:** 10.7759/cureus.49040

**Published:** 2023-11-19

**Authors:** Bayan Binsaeed, Fawaz G Aljohani, Faisal F Alsobiai, Maan Alraddadi, Alya A Alrehaili, Bushra S Alnahdi, Fahad S Almotairi, Mohammed A Jumah, Abdullah T Alrehaili

**Affiliations:** 1 Preventive Medicine, Ministry of Health, Macca, SAU; 2 Consultant Family Medicine, Ministry of Health, Madina, SAU; 3 General Practice, Ministry of Health, Riyadh, SAU; 4 Family Medicine, Ministry of Health, Madina, SAU; 5 College of Medicine, King Abdulaziz University Faculty of Medicine, Jeddah, SAU; 6 Nursing, Ministry of Health, Madina, SAU; 7 Consultant Family Medicine, Ministry of Health, Jeddah, SAU

**Keywords:** people with obesity, exercise, diet, barriers, motivators, intervention, weight loss, overweight, obesity

## Abstract

Introduction: Obesity has become a global public health challenge with associated health risks. Effective weight-loss interventions are crucial to mitigating these risks and improving overall well-being. However, individuals with obesity often encounter various barriers that hinder their weight loss efforts, while specific motivators can drive them towards successful outcomes. This systematic review aimed to explore the barriers and motivators to weight loss in people with obesity.

Methods: A literature search was conducted using relevant keywords in electronic databases such as PubMed, Medline, PsycINFO, and Google Scholar. Studies published in peer-reviewed journals during the last 10 years were considered for inclusion. We included studies investigating both barriers and/or motivators to weight loss published in English.

Results: The findings showed that motivators for weight loss include health concerns, body satisfaction, family support, normalcy restoration, emotional encouragement, self-determination, and mindful food choices. Motivators involve exercise facilities, balanced diets, and assistance from healthcare providers, peers, friends, or family. Gender influences healthcare providers’ influence on weight loss, with women trusting providers more while men lean towards medication options. Healthcare providers play a role in impacting weight loss through discussions and educating patients. Age also influences motivators; adolescents emphasize health, self-esteem, and bullying avoidance, while young women focus on lifestyle influence, resources, and joy. Barriers include insufficient self-control, physical pain, time constraints, dietary restrictions, and a lack of support. Logistical issues, patient readiness, healthcare providers' views, resource scarcity, and social dynamics are also barriers. Dietary barriers involve triggers, emotional states, and limited healthy options. School-aged children with obesity face curriculum challenges and resource scarcity.

Conclusion: These findings show the intricate interplay between motivators and barriers, underscoring the multifaceted nature of weight loss in people with obesity. Targeted interventions that address these factors holistically are essential for successful weight management.

## Introduction and background

Obesity and overweight are characterized by excessive fat accumulations that pose a health concern, with a body mass index (BMI) of 30 or above and 25 or higher, respectively [1]. Obesity has emerged as a global public health concern with significant implications for both individual well-being and healthcare systems, and its prevalence has risen steadily over the past few decades [2]. By 2016, obesity prevalence had reached 38%, and it is expected to reach 51% by 2030 in the United States of America alone, with overweight prevalence reaching 64.2% [2,3]. Globally, overweight and obesity were reported to affect 39% of adults in 2016, which is anticipated to rise to 58% by 2030 [4]. Effective weight-loss measures are critical for reducing the health risks associated with obesity, such as cardiovascular disease, diabetes, and some malignancies [5]. While weight loss measures frequently emphasize dietary adjustments, increased physical activity, and behavioral changes, sustaining long-term weight loss remains challenging [6]. This is partly due to the wide range of obstacles that people who are obese face on their weight loss journeys, which influence an individual's ability to begin and sustain weight loss efforts [7-9]. Lifestyle change is frequently recommended as the primary approach, as it carries a reduced likelihood of unfavorable outcomes compared to pharmacological and surgical interventions [10]. Nevertheless, altering behavior is widely acknowledged as a demanding endeavor, given the intricate nature of obesity, which encompasses other contributing physiological, psychological, social, and environmental factors [4,10]. Due to the complexity of the path to weight loss success, which is influenced by multiple factors that can either impede or facilitate development, understanding these factors, particularly the barriers experienced by individuals with obesity, is crucial for developing tailored and effective interventions to support weight management efforts [4]. Recognizing the motivators that lead obese people to embark on weight loss journeys can provide essential insights into how to support long-term commitment and adherence, and it can assist healthcare practitioners and researchers in developing interventions that are in line with individuals' personal goals and support the adoption of healthier behaviors. Therefore, this systematic review aimed to comprehensively provide a holistic understanding of the barriers and motivators to weight loss in people with obesity, which may inform evidence-based interventions. This aim will be achieved by answering the following research question: What are the barriers and motivators to weight loss in people with obesity?

## Review

Methods

Search Strategy

The primary research question for this systematic review was: What are the barriers and motivators to weight loss in people with obesity? A comprehensive literature search was conducted across electronic databases, including PubMed, Medline, PsycINFO, and Google Scholar. The search strategy incorporated a combination of keywords and MeSH terms, such as "obesity," "overweight," "weight loss," "barriers," "motivators," "factors," "incentives," "facilitators," and "interventions." The search encompassed peer-reviewed studies published in English between 2013 and 2023. The inclusion of this timeframe ensures relevance while considering recent developments. 

Inclusion and Exclusion Criteria

We selected studies that looked at both barriers and motivators, including adolescents and adults (aged 13-65 years) with obesity, regardless of ethnicity or gender. We included cross-sectional, cohort, case-control, randomization, experimental, meta-analysis, qualitative, and mixed-methods studies. Studies were excluded if they were case reports, reviews, opinions, and other non-original research articles. Moreover, studies concentrating solely on surgical treatments, weight reduction drugs, or pediatric populations were excluded.

Study Selection and Data Extraction

**Figure 1:**
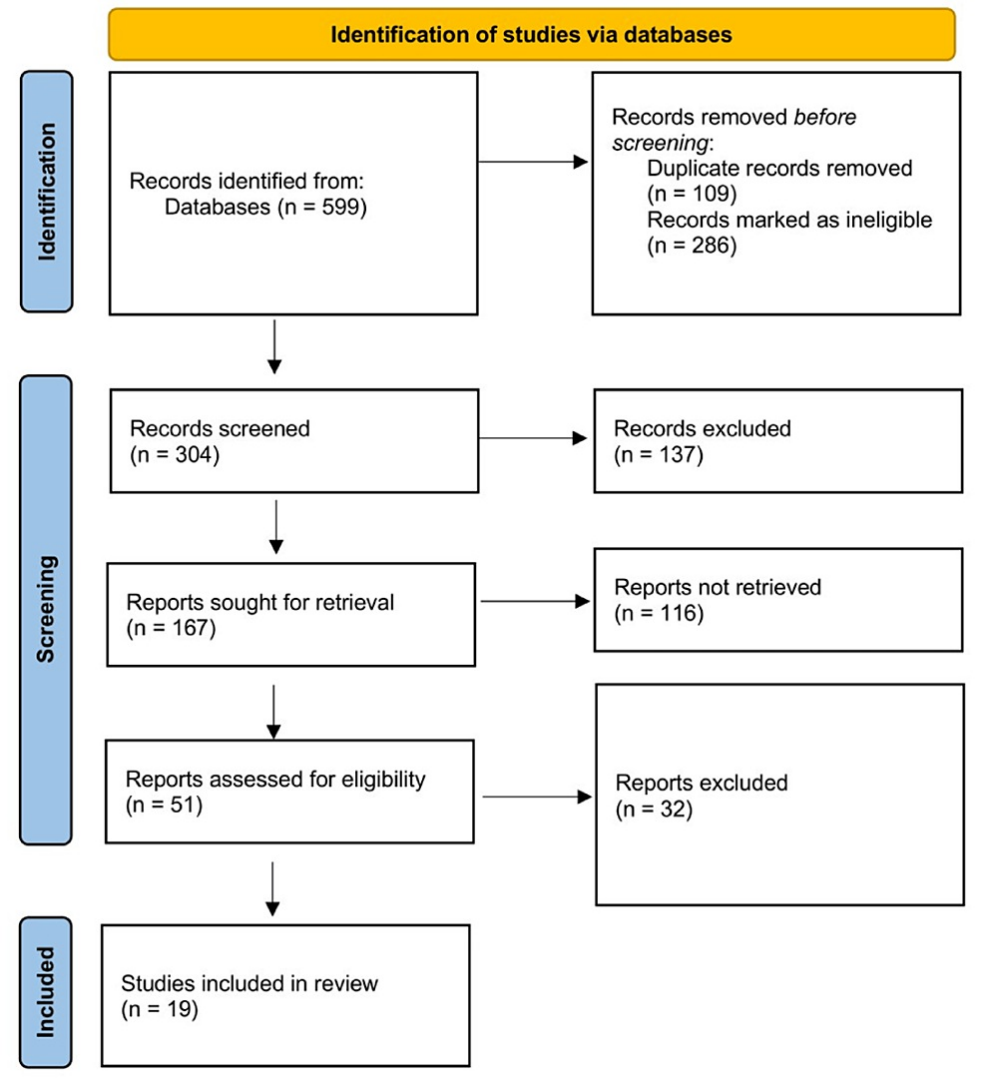
PRISMA flow diagram showing the selection process

Four independent reviewers first reviewed the search results by comparing titles and abstracts to the inclusion criteria. Any differences were worked out through debate and consensus. Following that, full-text papers from possibly qualifying articles were thoroughly reviewed. The study selection procedure was depicted using the Preferred Reporting Items for Systematic Reviews and Meta-Analyses (PRISMA) guide, as shown in Figure 1.

A standardized form was used during data extraction to obtain essential information from each selected study. Study features (e.g., authors, publication year, study design, and key findings) were all extracted. This procedure provided uniformity and made further analysis easier.

Quality Assessment

The quality assessment of the included studies was conducted to evaluate the robustness of the evidence and potential sources of bias. Different tools were utilized depending on the study design. For quantitative studies, the Newcastle-Ottawa Scale [11] for observational studies and the Cochrane Risk of Bias tool (CCRB) [12] for randomized controlled trials were employed. The Newcastle-Ottawa Scale, graded on a scale ranging from 0 to 9, is employed to assess the quality of cohort and cross-sectional research. It assesses aspects like the selection of participants, the comparison of study groups, and the evaluation of outcome or exposure. Each component of the Newcastle-Ottawa Scale, except for comparability, which allows for a two-point allocation, can attain only one point, yielding a minimum score of zero [11]. The Cochrane Collaboration's Risk of Bias tool encompasses seven dimensions: random sequence generation, allocation concealment, blinding of participants and personnel, blinding in outcome assessment, completeness of outcome data, selective reporting, and the potential presence of additional biases [12]. For qualitative studies, the Critical Appraisal Skills Program (CASP) checklist [13] was used. Five reviewers rated studies based on criteria such as sample representativeness, methodological rigor, and potential sources of bias.

Data Synthesis and Reporting

A thematic-synthesis approach was adopted to categorize the identified barriers and motivators. Themes were derived through an iterative process of data coding and thematic development. Given the heterogeneity across studies, a narrative synthesis was deemed more appropriate than a meta-analysis. Narrative synthesis refers to a method for conducting a systematic review and synthesis of findings from multiple studies, relying on the use of words and text to summarize and explain the synthesis's findings. While narrative synthesis may involve statistical data manipulation, its distinguishing feature is that it uses a textual approach to the synthesis process to 'tell the story' of the findings from the included studies. Therefore, we employed a narrative synthesis approach, summarizing extracted data to provide an overview of the barriers and motivators to weight loss in people with obesity. The findings of this systematic review were reported in a structured manner, including a description of the selected studies and identified barriers and motivators. 

Results

The initial search generated 599 results, and after removing duplicates and other articles according to exclusion criteria, 304 titles and abstracts were examined. The full-text versions of 167 studies were retrieved from qualifying titles and abstracts. The titles, abstracts, and full texts of 45 papers were deemed eligible and underwent a thorough review, which generated 19 articles fulfilling all inclusion criteria (Table 1). The majority of the included articles (10) were qualitative studies, four articles were cross-sectional studies, two articles were systematic reviews, and the other three articles were descriptive phenomenological studies, comparative trials and mixed methods studies.

**Table 1:** Characteristics of the included studies PCOS: Polycystic ovarian syndrome; KSA: Kingdom of Saudi Arabia

Authors	Year	Title	Study design	Summary of findings
Surrow et al. [14]	2021	The motivation and opportunities for weight loss related to the everyday life of people with obesity	A descriptive phenomenological study	Patients with overweight or obesity are driven to pursue weight loss due to health concerns and a desire for improved body satisfaction. The potential avenues for achieving weight loss involve establishing patterns, incorporating routines, and organizing daily activities to promote a healthier way of living.
Baillot et al. [15]	2021	Physical activity motives, barriers, and preferences in people with obesity: A systematic review	A systematic review	Weight control, increased energy/physical well-being, and social encouragement were the most common motives for physical exercise. The most prevalent obstacles were insufficient self-control/motivation, physical pain or discomfort, and time constraints. Walking emerges as the favored mode of physical activity for individuals with obesity.
Chew et al. [4]	2022	Personal motivation, self-regulation barriers and strategies for weight loss in people with overweight and obesity: a thematic framework analysis	Qualitative with a thematic framework analysis	Participants were primarily motivated to shed weight due to their role as a cornerstone of support for their family and a desire to regain a sense of normalcy. Barriers included overeating catalyzed by engagement in triggering activities (such as using social media); communal eating experiences with family, friends, and colleagues; food provision by others; emotional states (e.g., boredom, sadness, stress); physiological conditions (e.g., premenstrual syndrome); and specific times of the day.
Borazjaniet al. [8]	2022	Perceived Barriers to Weight Loss: A Qualitative Study of the Lived Experiences of Women with Obesity in Shiraz	Qualitative study	The participants recognized that dietary obstacles constituted unpleasant food taste, challenges in achieving satiety due to dietary restrictions, concerns about the sustained efficacy of the diet, its difficulty to adhere to, and experiencing diet-related side effects. Moreover, the absence of accessible social facilities and the general lack of societal awareness about obesity were identified as factors that could lead to the discontinuation of dieting efforts. Emotional support and encouragement from family and friends emerged as motivating factors. Engaging in gym sessions and procuring recommended dietary items were too expensive, hindering weight loss.
Dicker et al. [16]	2021	Patient motivation to lose weight: Importance of healthcare professional support, goals, and self-efficacy	Cross-sectional study	Recognition of the responsibility of healthcare professionals (HCPs) to contribute to weight loss (OR= 2.32, CI: 1.86–2.88), feeling at ease discussing weight matters with their HCP (OR= 1.46, CI: 1.24–1.72), agreement with the perception that weight loss is manageable (OR = 1.73, CI: 1.30–2.31), and having the objective of reducing health risks associated with excess weight (OR = 1.45, CI: 1.22–1.73) were associated with weight loss. Participants with obesity who considered obesity less significant than other health conditions were less motivated (OR = 0.49, CI: 0.41–0.58). Among people with obesity who expressed motivation to lose weight, those who exercised five times or more per week were more likely to lose weight compared to those who exercised less than once a week (OR = 2.77, CI: 2.09–3.68).
Coe et al. [17]	2017	Motivators, Barriers, and Facilitators to Weight Loss and Behavior Change Among African American Adults in Baltimore City: A Qualitative Analysis	A qualitative study	Motivators included the desire to achieve good health, inner drive and self-determination, a concern for longevity and quality of life, seeing results from weight loss efforts, and the potential for improved physical intimacy. Barriers included health concerns and physical limitations, seeking immediate versus delayed gratification, a lack of interpersonal support, unhealthy cultural norms, time constraints, cost, the disproportionate availability of unhealthy to healthy food options, a lack of community support, and transportation.
Silva et al. [18]	2018	Motivations for weight loss in adolescents with overweight and obesity: a systematic review	Systematic review	For adolescents, the most common motivations were better health, esthetic/cosmetic reasons, improvements in self-esteem, and avoidance of provocation or bullying.
Susanto et al. [19]	2023	Motivations for participation in weight loss clinical trials	Qualitative study	Male participants were motivated by healthcare professionals' recommendations to address weight concerns in order to prevent obesity-related coexisting conditions. On the other hand, family expectations and aesthetic considerations (such as clothing and fashion preferences) significantly motivated women to take part in weight-loss clinical trials.
Sand et al. [20]	2017	Motivation and obstacles for weight management among young women – a qualitative study with a public health focus - the Tromsø study: Fit Futures	Qualitative study with semi-structured in-depth interviews	Participants aimed for improved physical activity, diet, and routines. Parental influence on lifestyle was highlighted. Positive social settings and joy-motivated physical activity. The adulthood transition posed challenges to habits due to structural changes. More nutrition information was desired, and cost barriers to healthy food and sports were prevalent.
Trujillo-Garrido et al. [21]	2022	Motivation and Limiting Factors for Adherence to Weight Loss Interventions among Patients with Obesity in Primary Care	Cross-sectional study	A lack of adequate motivation for adhering to weight loss programs was reported by 67.5% of the participants. Only 3% of the assessed clinical records indicated documented behavioral change efforts. Patients commonly cited barriers to following diet and exercise plans, including an absence of a prescribed diet (27.8%), joint discomfort (17.7%), becoming fatigued or disinterested in dieting (14.8%), and feelings of laziness (11.5%).
Alick et al. [22]	2023	Motivating Weight Loss Among Black Adults in Relationships: Recommendations for Weight Loss Interventions	Cross-sectional study	Among those who had recently lost weight, personal factors such as significance and consciousness regarding health and self-awareness were recognized as individual facilitators that prompted the initiation of weight loss efforts.
Almubark et al. [23]	2019	Gender Differences in the Attitudes and Management of People with Obesity in Saudi Arabia: Data from the ACTION-IO Study	Cross-sectional study	The primary motivations for weight loss were to enhance their appearance and to seek increased energy levels. Females demonstrated greater trust in their healthcare provider's advice on weight management compared to males (87% vs. 82%) (p = 0.059), and they also expressed greater concerns about the long-term safety of prescription weight loss medications (65% vs. 59% males) (p=0.043). Males were more inclined to request their physician to prescribe newly introduced weight loss medications (p = 0.014), believed in the availability of effective weight loss medication options (p = 0.040), and exhibited a stronger preference for weight loss medications over surgical interventions (65% vs. 59%) (p = 0.054).
Metzgar et al. [24]	2015	Facilitators and barriers to weight loss and weight loss maintenance: a qualitative exploration	Comparative trial	Women perceived being answerable to others, receiving social assistance, preplanning, recognizing and being mindful of food selections, obtaining basic nutrition knowledge, managing portion sizes, engaging in physical activity, and fostering self-drive as essential facilitators for weight loss and weight loss maintenance. Barriers included life changes, alterations in health status, internal influences, external pressures, a lack of accountability, and the insufficiency of social support.
Al-Mohaimeed et al. [9]	2017	Experiences of Barriers and Motivators to Weight-Loss among Saudi People with Overweight or Obesity in Qassim Region - A Qualitative Study	Qualitative study	The primary triggers for weight loss are centered on health apprehensions and a wish to enhance their appearance. Inadequate familial encouragement and unhealthy eating habits during social events were identified as the predominant barriers. Factors promoting motivation included health worries, familial backing, and the presence of exercise facilities.
Helland et al. [25]	2021	Dietary Changes, Motivators, and Barriers Affecting Diet and Physical Activity among Overweight and Obese: A Mixed Methods Approach	A Mixed Methods Approach	Participants improved their eating habits following the initiation of exercise. After 33 weeks, the non-training group (NTG) consumed significantly more vegetables (p = 0.026) and legumes (p < 0.01) compared to the training group (TG). However, no significant differences were observed after one year of follow-up. Enhancing overall health emerged as the chief incentive for altering dietary and exercise habits. Hindrances to dietary changes included work commitments, family obligations, meal sizes, and internal decisions to modify behaviors. Time limitations and holiday periods were identified as barriers to exercise. Effective strategies included planned shopping and consistent physical activity for accomplishing and sustaining weight loss goals.
Tettero et al. [26]	2022	Barriers to and Facilitators of Participation in Weight Loss Intervention for Patients with Suboptimal Weight Loss after Bariatric Surgery: A Qualitative Study among Patients, Physicians, and Therapists	Qualitative study	Emotional reactions triggered by encountering unsatisfactory weight loss impeded patients' contemplation of engagement. Patients were open to their doctor's guidance in weight loss efforts if the physician acknowledged their independence. The utilization of visual weight loss charts proved effective in clarifying unsatisfactory weight loss for patients. Financial expenses and time limitations acted as barriers to participation.
Zevin et al. [27]	2019	Barriers to accessing weight-loss interventions for patients with class II or III obesity in primary care: a qualitative study	Qualitative study	Primary barriers included insufficient resource support, logistical challenges, limited knowledge about weight-loss interventions, patients' lack of readiness for change, family physicians' perceptions of surgical weight loss, and an incomplete comprehension of obesity's fundamental causes. Patients and physicians agreed on resource shortages, logistical issues, unfamiliarity with interventions, low motivation, and perceived surgical risks. They diverged on obesity's root causes, with physicians attributing it to multiple factors and patients emphasizing intrinsic causes. Both groups highlighted the importance of effectively addressing these barriers to enhance weight-loss interventions.
Lim et al. [28]	2019	Barriers and facilitators to weight management in overweight and obese women living in Australia with PCOS: a qualitative study	Qualitative study	Facilitators for weight loss included a balanced diet and receiving assistance from healthcare providers, peers, friends, or family. Challenges included logistical hindrances such as time and financial constraints, motivational impediments including fatigue or a lack of perceived rewards, environmental obstacles like limited access to safe exercise spaces, emotional barriers such as depressive and demotivating thoughts, and relational difficulties such as an unsupportive partner or prioritizing children's dietary preferences.
Almutairi et al. [29]	2022	Barriers and enablers to the implementation of school-based obesity prevention strategies in Jeddah, KSA	Qualitative study	Respondents recognized five distinct groups of barriers to executing obesity prevention interventions: curriculum-related challenges, school strategies endorsing healthy weight, resource scarcity, students' lifestyles, and a lack of teachers specializing in nutrition and sports. On the other hand, facilitating factors frequently cited were school regulations, a capable staff, awareness, and resources.

One study reported that patients and doctors agreed on challenges like resource shortages, logistics, and low motivation for weight-loss interventions. They differed on obesity's causes yet stressed addressing barriers to effective treatment [27]. Regarding motivators for weight loss, health concerns and a desire for improved body satisfaction, the desire to support their family and a desire to regain a sense of normalcy, emotional support and encouragement from family, inner drive and self-determination, and preplanning, recognizing, and being mindful of food selections were the most common [4,8,9,14,15,17,24]. Moreover, facilitators included exercise facilities, a balanced diet, and assistance from healthcare providers, peers, friends, or family [9,28]. The role of healthcare providers in motivating patients with obesity to lose weight was also reported in more studies, and it was influenced by gender. One study that reported appearance and energy as motivators for weight loss found that women trusted healthcare providers more (87% vs. 82% of men) and were more concerned about medication safety (65% vs. 59%). Men leaned toward new medications (p = 0.014), believed in options (p = 0.040), and preferred medications over surgery (65% vs. 59%, p = 0.054). Women saw accountability, social support, planning, food choices, nutrition knowledge, portion control, activity, fashion, and self-motivation as vital for weight loss and maintenance [19,23]. Furthermore, recognition of the responsibility of healthcare professionals (HCPs) to contribute to weight loss (odd ratio [OR]= 2.32, confidence interval [CI]: 1.86-2.88) and feeling at ease discussing weight matters with their HCP (OR= 1.46, CI 1.24-1.72) positively impacted weight loss [16]. Attitudes and perceptions of people with obesity may also play a role in agreement that weight loss is manageable (OR = 1.73, CI: 1.30-2.31) and has the objective of reducing health risks associated with excess weight (OR = 1.45, CI: 1.22-1.73) [16].

Age seems to influence the motivators for weight loss. For adolescents, the most common motivations were better health, esthetic/cosmetic reasons, improvements in self-esteem, and avoidance of provocation or bullying; and for young women, parental influence on lifestyle, social settings, joy, school regulations, a capable staff, awareness, and resources were motivators [18,20,29]. Some effective strategies for weight loss include establishing patterns, incorporating routines and diets, and organizing daily physical activities [14,15,25]. The utilization of visual weight loss charts guided by healthcare providers proved effective [26]. It was found that physical exercise helped improve diet, leading to significant consumption of more vegetables (p = 0.026) and legumes (p < 0.01) after 33 weeks of exercise [25]. Of all measures for weight loss, walking emerged as the favored mode of physical activity, and those who exercised five times or more per week were more likely to lose weight compared to those who exercised less than once a week (OR = 2.77, CI: 2.09-3.68) [15,16]. 

Barriers to weight loss among people with obesity included insufficient self-control/motivation, physical pain or discomfort, time constraints, unpleasant food taste, challenges in achieving satiety due to dietary restrictions, concerns about the sustained efficacy of the diet, its difficulty in adhering to, experiencing diet-related side effects, life changes, alterations in health status, internal influences, external pressures, a lack of accountability, and insufficiency of social support [8,15,16,21,24,26]. Barriers encompassed resource shortages, logistical issues, patient readiness, physician views on surgery, limited obesity understanding, time and financial constraints (expensive healthy diet and gym subscription), motivation, environment, emotions, and relationships, affecting exercise, mental state, support, and dietary choices [27,28].

Regarding dietary changes, barriers included overeating catalyzed by engagement in triggering activities, such as using social media, communal eating experiences with family and friends, emotional states such as boredom, sadness, stress, and disproportionate availability of unhealthy to healthy food options, as well as physiological conditions such as premenstrual syndrome [4,9,17,29]. For school-aged patients with obesity, barriers included curriculum-related challenges, school strategies endorsing healthy weight, resource scarcity, students' lifestyles, and a lack of teachers specializing in nutrition and sports, as well as adolescence [8,20]. 

Discussion

Obesity's rising prevalence necessitates a better understanding of the elements that determine successful weight-loss efforts. This systematic review seeks to give a thorough picture of the barriers and motivators experienced by individuals with obesity. The findings of this research have the potential to allow the creation of focused interventions that address barriers and leverage motivators, thereby increasing the efficacy of weight loss programs and the long-term health outcomes of people with obesity.

This systematic review showed various motivators and facilitators for weight loss in individuals with obesity. These include health concerns, body satisfaction, family support, normalcy restoration, emotional support, self-determination, and mindful food choices. These findings align with previous studies [30,31]. Another previous systematic review showed that choosing a Mediterranean diet was associated with weight loss and maintenance [32]. This is attributed to the ingredients of the Mediterranean diet, which are rich in fruit and vegetables, making it healthy compared to the Western diet made of processed food [33,34]. We also found that exercise facilities and support from healthcare providers, peers, and family were facilitators. Healthcare professionals' involvement and open weight discussions positively impacted weight loss. Belief in manageable weight loss and reducing health risks are correlated with successful weight loss. Aligning with these findings, Alfadda et al. [31] found that both people with obesity and healthcare providers were responsible for weight loss. In their study, people with obesity expressed a sense of responsibility for managing their weight (67%), while healthcare providers recognized their role in contributing to this effort (71%). In total, 58% of people with obesity engaged in weight-related discussions with their healthcare providers within the past five years, and 46% had received an official obesity diagnosis. Additionally, 44% had scheduled follow-up appointments. Although 50% of people with obesity indicated a motivation to shed weight, only 39% of healthcare providers believed their patients were genuinely motivated to do so. When it came to genetic factors as a barrier to weight loss, fewer than half of people with obesity (39%) and healthcare providers (49%) considered them significant [31]. Further studies also reported that healthcare providers can positively influence people with obesity to engage in weight loss efforts and succeed [35,36]. Healthcare providers can guide patients, as our systematic review showed that guiding charts from healthcare providers were motivators for weight loss. Charts allow patients to break down weight loss goals into smaller, more achievable targets, enhancing motivation and perceived self-efficacy. Additionally, healthcare providers educate patients and help remove the stigma and weight bias associated with overweight and obesity, leading to successful weight-loss interventions [37]. A study by Kaplan et al. [38] reported that despite several weight loss attempts, only 23% of people with obesity reported 10% weight loss during a three-year period. Most (65%) recognized obesity as a disease, but only 54% worried their weight might affect their future health. Most people with obesity (82%) felt completely responsible for weight loss, while 72% of healthcare providers felt responsible for contributing to weight loss efforts [38].

This systematic review showed that gender influenced motivators to weight loss, with women valuing trust, safety, and accountability, while men leaned towards medication options, aligning with a previous study by Salem et al. [32]. Previous studies also found that gender is a factor in weight-loss interventions. For example, one prospective cohort study found that while men and women can achieve weight loss following weight loss therapy, women are more likely to achieve better results [39]. This might be explained by the report by another study that men were discouraged from engaging with or continuously attending sessions due to gender imbalance and attitudes toward existing weight loss services [40]. Moreover, previous studies found that women were more likely to engage in weight loss attempts and compare their appearances to others, which prompted them to engage in weight loss efforts [41], aligning with our findings. Our systematic review also showed that for adolescents and school-aged children with obesity, the motivators for weight loss were different from those among adults. The most common motivations were better health, esthetic/cosmetic reasons, improvements in self-esteem, and avoidance of provocation or bullying. For young women, parental influence on lifestyle, social settings, joy, school regulations, a capable staff, awareness, and resources were motivators. Previous studies showed that children with obesity are more likely to be victims of bullying and have less self-esteem [41-43], which explains these motivators in addition to the adolescent period that increases image obsession [44]. However, this body image obsession was found to lead to extreme weight loss measures that are unhealthy and sometimes dangerous and can also cause eating disorders [45,46]. Our study found that effective weight loss strategies included consistent physical exercise, and walking was favored. Though one study in our systematic review showed that the non-training group (NTG) consumed significantly more vegetables (p=0.026) and legumes (p < 0.01) compared to the training group (TG) at 33 weeks, this difference was insignificant at one year. Overall, we found that physical exercises helped improve diet, leading to significant consumption of more vegetables (p = 0.026) and legumes (p < 0.01), ingredients of a healthy diet, which explains the reason more frequent exercise was linked to greater weight loss success. Physical exercises and walking, in particular, are also well-known weight-loss measures proven effective in the literature [40,43,47,48].

Barriers to weight loss included insufficient motivation, physical discomfort, time constraints, diet restrictions, and doubts about sustainability. One previous study found that time constraints and a lack of motivation were barriers to physical activity, one of the most effective measures for weight loss [49]. Moreover, this study showed that lack of time, convenience, and knowledge on how to make healthy food choices were barriers to eating healthier, limiting weight loss success [49]. Previous studies also showed that busy schedules, lack of time for meal preparation and exercise, emotional eating, stress, depression, low self-esteem, family responsibilities, cultural norms, and lack of social support were identified as significant barriers [6,50,51], consistent with our findings. Another previous study showed that, for children with obesity, an unemployed father (OR=11.90; 95% CI: 7.47-18.93), a father with overweight or obesity (OR=2.04; 95% CI: 1.40-2.96), an incorrect parental perception of the child’s weight status (OR=2.54; 95% CI: 1.75-3.68), physical activity for less than 30 min (OR=2.18; 95% CI: 1.44-3.28), frequent snacking (OR=1.74; 95% CI: 1.05-2.93), and screen time use for more than 2 hours per day outside of school (OR=1.62; 95% CI: 1.12-2.34) were associated with weight gain and unsuccessful weight loss efforts [52]. This is similar to our findings that overeating due to triggers such as social media, emotions, and unhealthy food availability were barriers to weight loss among school-aged children with obesity. There is evidence that mood disorders (depression, anxiety, and low self-esteem) and eating disorders (binge eating disorder) are associated with poor weight loss among people with obesity [45,53,54]. For students with obesity, challenges included school-related issues, resource scarcity, and adolescent factors, including eating disorders prevalent in adolescence. In addition, barriers, such as school strategies endorsing healthy weight, students' lifestyles, and a lack of teachers specializing in nutrition and sports, were highlighted by our findings. These findings indicate that schools and education authorities should establish measures to allow students to practice healthier lifestyles, especially those with obesity. Research indicates that schools have made certain progress in enhancing healthier food and physical activity settings over the last decades. However, substantial further efforts are required [55]. Therefore, enhanced policies are essential to provide students with healthier meals, restrict the availability of high-calorie foods during school hours, and amplify the frequency, intensity, and duration of physical activities within schools. Moreover, research has indicated the effectiveness of implementing school-based nutrition and physical activity policies to prevent obesity and overweight among students [56]. We found that emotional reactions triggered by encountering unsatisfactory weight loss were a barrier to weight loss, consistent with other studies showing that unrealistic expectations, confusion, and frustration were barriers to weight loss [57]. The weight-loss journey is long and often stressful, requiring a high level of discipline to succeed. Therefore, people on this journey are susceptible to doubting their ability to achieve weight loss goals, hindering their motivation and efforts and contributing further to failure [58]. Therefore, it requires self-motivation and support from friends, family members, and healthcare providers. Though healthcare providers reported willingness and comfort with weight-related conversations with patients, it was found that time constraints often deprioritized these efforts [38]. Another study found that 58% of people with obesity engaged in weight-related discussions with their healthcare providers within the past five years. However, although 50% of people with obesity engaged in conversations that indicated a motivation to lose weight, only 39% of healthcare providers believed their patients were genuinely motivated to do so [31]. It was suggested that the support from employers of people with obesity is also effective, but one study found that only 17% of people with obesity perceive employer-sponsored healthy lifestyle interventions as helpful in supporting weight loss [38].

There are some limitations we should consider for this systematic review. First, relying on peer-reviewed and published articles could lead to publication bias since studies with positive outcomes are more likely to be published. The comparability is limited by the variability in methodologies used by the different included studies. Variations in cultural, socioeconomic, and healthcare system contexts among studies could also limit generalizability. 

Despite those limitations, this systematic review can have implications in different ways. By understanding motivators and barriers to weight loss, comprehensive strategies in healthcare and educational settings can be taken or improved to enhance the effectiveness of weight loss programs. The findings of this study could also help establish a holistic approach involving economic and emotional support and other advocacy measures to ensure weight loss programs succeed, potentially improving long-term adherence and success rates. This systematic review highlights the complexity of motivators and barriers to weight loss. It can work as a baseline for future research to explore specific demographic groups, evolving trends, and the effectiveness of emerging interventions.

## Conclusions

This systematic review showed that motivations for weight loss include health concerns, body satisfaction, family support, and regaining normalcy, driven by inner determination and planning. Exercise facilities, balanced diets, and healthcare assistance aid in weight loss. Recognizing healthcare professionals' role positively impacts weight loss. Positive attitudes and perceptions, like manageable weight loss and reducing health risks, correlate with success. On the other hand, barriers included low motivation, discomfort, time constraints, dietary challenges, sustainability doubts, and a lack of support. Constraints ranged from finances and environment to emotions and relationships, impacting exercise and dietary choices. Dietary change barriers included triggers like social media, emotions, and junk food availability. The findings also showed that gender and age modulate both barriers and motivators to weight loss, highlighting the need for targeted interventions in consideration of gender and age to address barriers and leverage motivators, ultimately enhancing the effectiveness of weight-loss strategies and improving long-term health outcomes among people with obesity.
